# Quantity–quality trade‐offs revealed using a multiscale test of herbivore resource selection on elemental landscapes

**DOI:** 10.1002/ece3.6975

**Published:** 2020-11-18

**Authors:** Juliana Balluffi‐Fry, Shawn J. Leroux, Yolanda F. Wiersma, Travis R. Heckford, Matteo Rizzuto, Isabella C. Richmond, Eric Vander Wal

**Affiliations:** ^1^ Department of Biology Memorial University of Newfoundland St. John’s NL Canada; ^2^ Present address: Department of Biological Sciences University of Alberta Edmonton AB Canada

**Keywords:** ecological stoichiometry, foraging ecology, landscape ecology, moose, resource selection analysis, scale

## Abstract

1. Herbivores consider the variation of forage qualities (nutritional content and digestibility) as well as quantities (biomass) when foraging. Such selection patterns may change based on the scale of foraging, particularly in the case of ungulates that forage at many scales.
2. To test selection for quality and quantity in free‐ranging herbivores across scales, however, we must first develop landscape‐wide quantitative estimates of both forage quantity and quality. Stoichiometric distribution models (StDMs) bring opportunity to address this because they predict the elemental measures and stoichiometry of resources at landscape extents.
3. Here, we use StDMs to predict elemental measures of understory white birch quality (% nitrogen) and quantity (g carbon/m^2^) across two boreal landscapes. We analyzed global positioning system (GPS) collared moose (*n* = 14) selection for forage quantity and quality at the landscape, home range, and patch extents using both individual and pooled resource selection analyses. We predicted that as the scale of resource selection decreased from the landscape to the patch, selection for white birch quantity would decrease and selection for quality would increase.
4. Counter to our prediction, pooled‐models showed selection for our estimates of quantity and quality to be neutral with low explanatory power and no scalar trends. At the individual‐level, however, we found evidence for quality and quantity trade‐offs, most notably at the home‐range scale where resource selection models explain the largest amount of variation in selection. Furthermore, individuals did not follow the same trade‐off tactic, with some preferring forage quantity over quality and vice versa.
5. Such individual trade‐offs show that moose may be flexible in attaining a limiting nutrient. Our findings suggest that herbivores may respond to forage elemental compositions and quantities, giving tools like StDMs merit toward animal ecology applications. The integration of StDMs and animal movement data represents a promising avenue for progress in the field of zoogeochemistry.

## Graphical abstract

We use novel stoichiometric distribution models (StDMs) to test if moose follow scale‐dependent forage quality and quantity expectations across a forested landscape in Newfoundland, Canada. Using elemental measures for forage quantity and quality as our only two explanatory covariates, and three scales of resource selection analyses, we were able to explain variation in resource selection by individual moose. We found no scalar pattern, counter to our prediction, but we uncovered divergent, individual‐based quantity–quality trade‐off tactics.

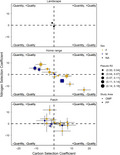

## 1 INTRODUCTION

Finite energy and material within ecosystems forces constraints upon all trophic levels. Heterotrophs are left to optimize their energy intake with strategic foraging and evolved digestive tracts (Pyke et al., 1977; Werner & Hall, 1974). While producers consist mostly of carbon‐based compounds, consumers consist of proportionally more nitrogenous and phosphorous compounds. Thus, primary consumers must eat relatively large amounts of producer matter to meet their body composition requirements (Barboza et al., 2009; Fagan et al., 2002; Sterner & Elser, 2002). Additionally, access to plant matter of higher digestibility and assimilation efficiency can contribute to higher animal growth rates, survival, and reproductive outputs (McArt et al., 2009; Parker et al., 2009; Wam et al., 2018). As a result, herbivores have evolved strategies to forage with consideration to plant qualities (i.e., digestive efficiency) and quantities (i.e., biomass or abundance; Parker et al., 2009). Determining plant quality requires tissue composition analysis, limiting our ability to measure and map plant quality across landscapes. Large herbivores forage at multiple spatial scales, from the landscape to the bite‐level (Johnson, 1980; Senft et al., 1987), and likely respond differently to plant quantities and/or qualities across scales, collectively influencing their ecosystem effects (Estes et al., 2011; Schmitz et al., 2018).

Herbivore strategy for food acquisition may depend on the scale of foraging (Cruz‐rivera & Hay, 2000; Hebblewhite et al., 2008; Van der Wal et al., 2000; Wilmshurst et al., 1999). In terrestrial landscapes, plant biomasses and nutritional contents are influenced by environmental factors such as habitat type (Sardans et al., 2011), soil nutrients (Fan et al., 2015; Sardans et al., 2011), elevation (Yang et al., 2015), or slope (Leroux et al., 2017), creating a heterogeneous distribution of plant quantities and qualities. When plant quantity and quality do not positively correlate across a landscape, herbivores should adopt one of multiple strategies, such as balancing selection between quantity and quality or selecting one over the other. An individual's tactic for quantity and quality selection is likely to depend on scale because information available for decision‐making increases in resolution with reducing scales of foraging (Rettie & Messier, 2000; Senft et al., 1987). The scalar hypothesis predicts that coarser factors that affecting forage quantities, like climate, water bodies, and plant biomass, influence larger scaled foraging decisions, for example, home‐range selection (Bailey et al., 1996; Wilmshurst et al., 1999). Finer factors that influence smaller scaled foraging decisions, for example, patch use or bite choice, are often quality‐related indicators: plant morphology, palatability, nutrient content, and secondary compounds (Bailey et al., 1996; Senft et al., 1987; Verheyden‐Tixier et al., 2008; Wilmshurst et al., 1999). Thus, herbivores are likely to show selection for plant quantities at the larger scales of foraging and for plant quality at smaller scales (van Beest et al., 2010).

Testing selection for plant qualities across multiple scales of foraging remains challenging in many systems because measuring plant quality at larger spatial extents may not always be feasible. Plant biomass has been quantified across larger spatial extents in various ecosystems (Foroughbakhch et al., 2005; Fortin et al., 2003; Lone et al., 2014). Meanwhile, Weisberg and Bugmann (2003) highlight the need for an “accurate database of the spatial heterogeneity of available forage of varying quality, over the same fine scales as are modeled” (p. 4) as a way to measure quality in the context of foraging strategies for ungulates. However, due to landscape data limitations, studies remotely measuring ungulate responses to spatial distributions of forage usually default to habitat type or dominant plant species classifications as estimates of forage quality and subsequently disregard intraspecific variation in quality (for example van Beest et al., 2010). While using browse species is not an incorrect way to capture quality variation, it limits which foraging scales a study can investigate, findings may not be comparable across systems, and model responses to categorical variables cannot be directly compared to those of continuous variables.

Plant elemental compositions offer the opportunity to describe interspecific and intraspecific variation of forage quality in a continuous manner. Plant nitrogen content is a common elemental measure to correlate with forage quality because nitrogen is a limited nutrient in terrestrial ecosystems, required for protein synthesis, and needed in higher proportion by animals relative to plants (Fagan et al., 2002; Sterner & Elser, 2002). Elements are a base unit for all living organisms, and heterotrophs rearrange element components they consume into compounds they require. Thus, the direct nutritional driver behind foraging may best observed by measuring selection for nitrogen content rather than composite currencies or nutritional compounds (Felton et al., 2018). However, studies which measure herbivore responses to plant nitrogen contents often do so with small‐bodied herbivores (Schatz & McCauley, 2007), use smaller scales of observation (Nie et al., 2015), or are otherwise restricted to experimental conditions (Ball et al., 2000; but see Champagne et al., 2018, Moore et al., 2010). Certain technological developments, such as high‐resolution airborne imaging spectroscopy, have made landscape‐wide mapping of nitrogen content possible, but only in ecosystems where the forage is aerially visible (Schweiger et al., 2015). Recently developed methods, termed Stoichiometric Distribution Models (StDMs; sensu Leroux et al., 2017) present a solution by modeling understory plant elemental quantities and compositions across landscapes, allowing for variation in both forage quantities and qualities to be predicted across landscapes.

**Figure 1:**
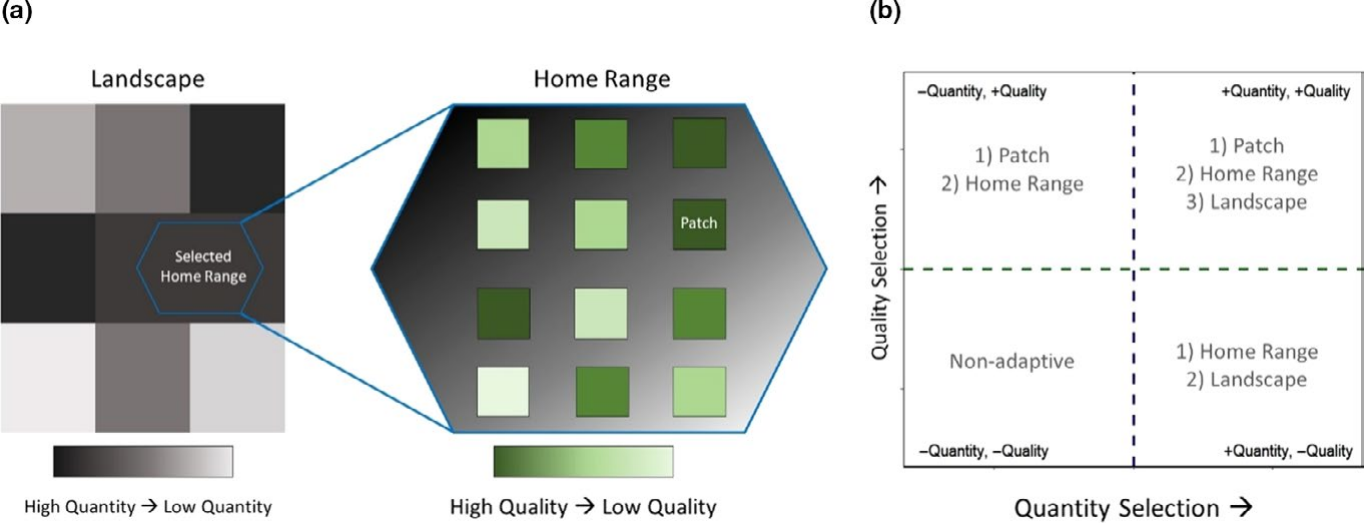
(a) Conceptual diagram of resource grain from the perspective of different spatial extents. Landscapes are composed of a coarse patchwork of forage quantities, within which are home ranges with a finer‐scaled gradient of quantities and patches of forage varying in both quantity and quality. (b) According to literature, at the landscape extent, herbivores should most often positively select for quantity, while at the patch extent, they should most often positively select for quality. At the home range extent, either quantity or quality could be selected for. At no scale should moose negatively select for both quantity and quality

Here, we used StDMs to investigate selection strategies of forage quantity and quality, across multiple spatial extents for a large, wide‐ranging, understory browsing mammal. We studied a moose (*Alces alces*)—white birch (*Betula papyrifera*) system on the island of Newfoundland, Canada. White birch grows widely and is heavily preferred by moose in Newfoundland (Dodds, 1960). We measured individual moose resource selection in relation to understory white birch availability at the landscape, home range, and patch scales. Our landscape‐wide estimates of available forage for white birch quantities and qualities derive from the continuous elemental predictions of the StDMs. Our objective was not to create highly predictive, cross‐season moose resource selection models, but to test the relationship between moose habitat selection patterns and distributions of browse in terms of browse elemental predictors for quantity and quality across multiple foraging scales. To do so, we measured moose resource selection during the short temporal window of the early growing season in Newfoundland as this was the temporal window of the StDM predictions. We predicted that if moose show selection for white birch quantity and quality, their selection for quantity would be highest at the landscape extent and decrease when refined to home range and then patch extents, while the reverse would be true for white birch quality selection (van Beest et al., 2010). We also predicted that quantity–quality trade‐offs may occur, but the direction of such trade‐offs would depend on the scale of foraging; at no scale should there be negative selection for both plant quantity and quality (Figure 1). Collectively, this study represents an opportunity to test the foraging strategies of an ungulate species under non‐experimental conditions by linking the biogeochemical landscape to herbivore movements.

## 2 METHODS

### 2.1 Study region and moose collaring

**Figure 2:**
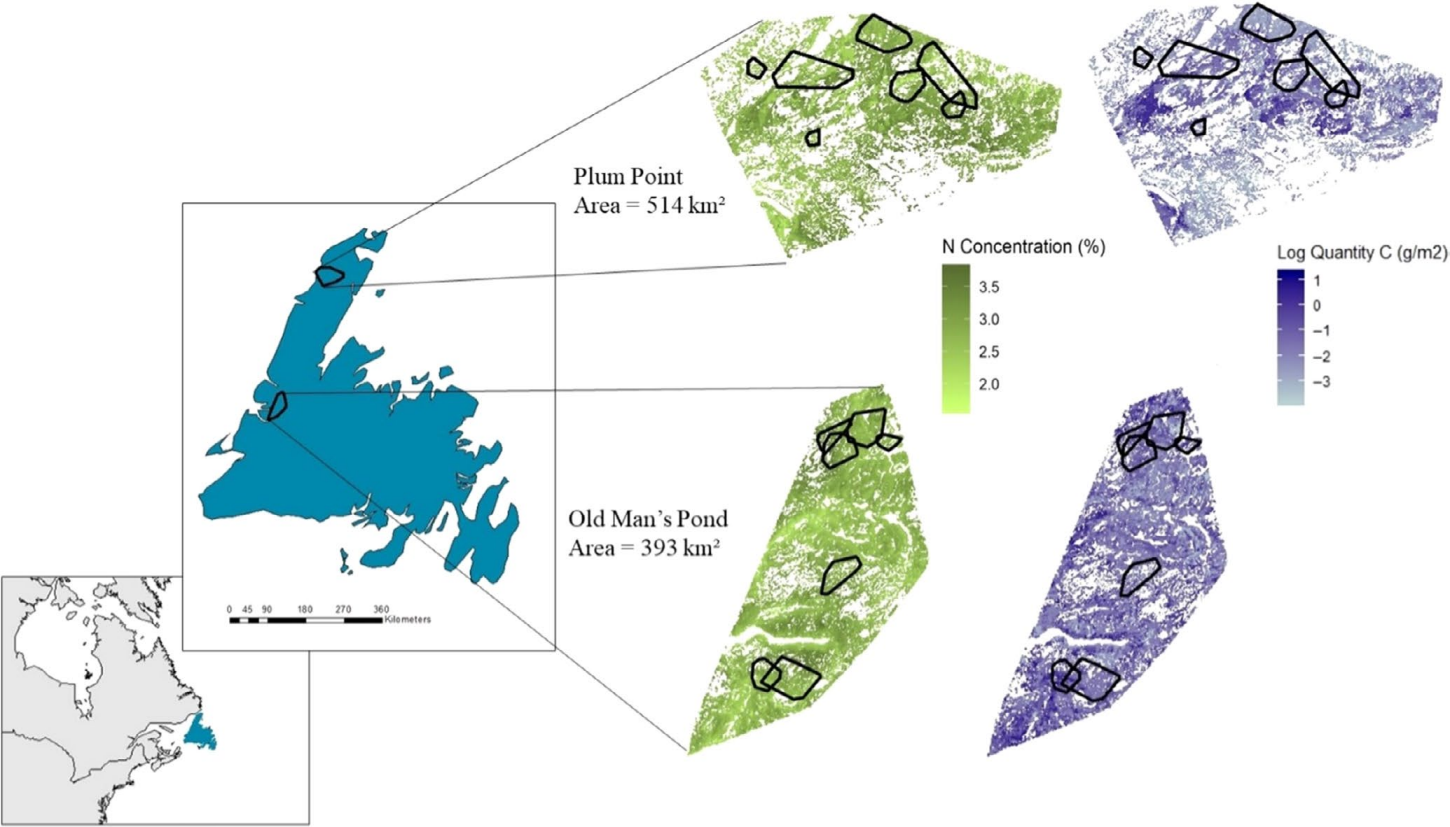
The island of Newfoundland in relation to eastern North America, with the boundaries of our study areas shown. Within each study area, we show their stoichiometric distribution model outputs for white birch forage nitrogen concentrations and carbon quantities and the MCPs of each study area's study moose home ranges. White areas are areas where we have no inference for certain habitat types like wetlands or water bodies. MCP, minimum convex polygons

This study took place on the northern peninsula of the island of Newfoundland, Canada (Figure 2). Dominant tree species of this region include black spruce (*Picea mariana*), balsam fir (*Abies balsamea*), and white birch, which is the primary forage for moose in Newfoundland during early summer (Dodds, 1960). We collared 14 adult moose (male = 4; female = 10) in this region between 2011 and 2015 with global positioning system (GPS) collars set to take locations every two hours. Each individual was collared for the duration of one year (2011 *n* = 5; 2013 *n* = 1; 2014 *n* = 5; 2015 *n* = 3; Table 2). The 14 moose collars were deployed in two areas approximately 300 km apart within the island of Newfoundland: Plum Point study area (PP; *n* = 7) and Old Man's Pond (OMP; *n* = 7; Figure 2). Three additional moose were collared in a third study area (Leroux et al., 2017), but we did not use these data because there were too few moose individuals to compare landscape‐scale selection with the other study areas. The boundaries of each study area were delineated using minimum convex polygons (MCPs) at 95% around all of their respective moose fixed locations (PP = 514 km^2^; OMP = 393 km^2^; Figure 2).

### 2.2 Forage quantity and quality measures

We used spatial predictions from StDMs, a method for predicting resource elemental compositions and quantities across a landscape, to represent forage resources in this study (Leroux et al., 2017). We clipped white birch leaves from the browsing heights (0.3–2.0 m) of 1–6 individuals at 10 m radius plots (*n* = 106) across the Plum Point study area. Sampling was constrained to a small temporal window (June 30 and July 7, 2015) representing green‐up time to minimize temporal variation in foliar elemental composition due to senescence. At each plot, we measured densities of three size classes of white birch by height (0.3–0.5 m; 0.51–1 m; 1.01–2 m). We estimated biomass for each age class by measuring standing stocks (all leaves between heights 0.3–2.0 m) from a sample of trees and then used these estimates to calculate total white birch biomass for each plot (Leroux et al., 2017). Ground‐collected samples were then sent to the Agriculture and Food Laboratory at University of Guelph (Guelph, Ontario, Canada) and analyzed for carbon, nitrogen, and phosphorus compositions (%). Using the biomass estimates and elemental compositions, we calculated elemental quantities (g/m^2^) for each plot. Lastly, the carbon, nitrogen, and phosphorous quantities (g/m^2^) and compositions (%) of newly developed understory white birch growth (June 1st–July 16th) were fit to six landscape predictors across the two study areas of our moose collar data (Leroux et al., 2017; Table 1). Landscape predictors included three abiotic features—normalized aspect, slope, and elevation—and three biotic features—landcover, stand height, and dominant tree species (see Table 1 for StDM covariate details). Because our plant data derives from StDMs fit for the specific temporal window of early summer, we subset all collar data to only include fixes from that same temporal window (June 1st–July 16th). There was a mismatch of year between some individual moose GPS collar data and forage data from StDMs (1–4 years, mean = 1.93). The six‐explanatory landscape variables that predicted forage elemental measures in the StDMs are fairly static in this system relative to the 4‐year window of mismatch. We assume the relative StDM predictions to remain consistent within this 4‐year window (i.e., areas with high quantity of white birch in year t will also have high quantity in year t + 1), but also assume moose response findings to be conservative given inter‐annual climatic variation.

**Table 1:** Explanatory covariates used in the stoichiometric distribution models to predict the white birch quantity carbon and nitrogen composition values, the type of data each covariate provided, and the description of each covariate's calculation or categories

Predictor variable	Data type	Description/categories
Normalized aspect	Continuous	Direction of slope
Slope	Continuous	Tangent of surface angle to horizontal
Elevation	Continuous	Height above sea level
Landcover	Categorical	Two categories: Coniferous and other (deciduous or mixed wood)
Stand Height	Categorical	Four categories: 0–6.5 m; 6.6–9.5 m; 9.6–12.5 m; 12.6–21.5 m.
Dominant Tree Species	Categorical	Three categories: 75% balsam fir; 50%–75% balsam fir with remainder black spruce and/or white birch; 50–75 black spruce or white spruce with remainder balsam fir white birch, or tamarack.

We used final StDM spatial predations of reasonably strong fits (*R* ^2^). We used StDM predictive surfaces of white birch carbon quantity (log g/m^2^; *R* ^2^ = 0.28) to represent forage quantity. To estimate forage quality, we used StDM predictive surfaces of white birch nitrogen composition (%; *R* ^2^ = 0.31) and assume nitrogen concentration to positively correlate with browse quality (Ball et al., 2000; Mattson, 1980; McArt et al., 2009). The StDMs also predicted birch elemental ratios, elemental ratios are calculated from elemental compositions and quantities, so we used nitrogen composition rather than carbon:nitrogen as our proxy of forage quality to maintain an independence between quantity and quality.

While elemental currencies do not account for plant secondary metabolite (PSM) concentrations, moose have been found to be nitrogen‐constrained (McArt et al., 2009). *Betula* species favor using carbon‐based PSMs (Palo, 1984), have also been experimentally shown to be nitrogen‐limited, and display most PSMs at higher concentrations under greater UV exposure rather than greater nitrogen fertilizer (Keski‐Saari et al., 2005). Since plants often acquire fiber and lose nutritional content as they gain biomass during the growing season, (Hebblewhite et al., 2008), we also tested for negative correlations (Pearson's *r*) between our white birch quantities and qualities which could be driven by static, landscape variation in plant age.

### 2.3 Defining the scales of foraging

We examined moose resource selection at the landscape, home range, and patch extents, or second, third, and 3.5th order selection according to the Johnson (1980) framework. We used the R statistical program for all analyses (version 3.5.1; R Core Team, 2018). We designated each study area, PP or OMP, to be the “landscape” for its moose individuals. We calculated “home range” extents for each individual with MCPs at 95% around all GPS fixes within the time window of this study (June 1st–July 16th) using the R package “adehabitatHR” (Calenge, 2006). Lastly, we defined our highest resolution of forage landscape data, 30 m × 30 m pixels, to be “patches,” or the immediate area around a sample or GPS fix point. We did not collect plant data in waterbodies, wetlands, roads, etc., and did not use StDMs to predict forage values in these habitats (Leroux et al., 2017). Therefore, we cleaned all resource selection analysis data, including moose GPS data, to only include points within forested areas with StDM predictions. We used two types of resource selection analyses and the same predictive StDM landscape layers to test selection at all three scales (Figure 1; Figure S1).

### 2.4 Forage selection: landscape extent

At the landscape, our study's largest spatial extent, foraging decisions include where an animal places its home range (Boyce, 2006; Johnson, 1980). To test if home ranges differ in the availability of forage quantities and qualities compared to the landscape, we used a resource selection function (RSF), a model which compares used and available locations of an organism and can be fit with logistic regression by assuming the exponential function:

$$w\left(x\right)=\exp\left(\beta_{0}+\beta_{1}x_{1}+\beta_{2}x_{2}+\cdots +\beta_{k}x_{k}\right)$$

with *x_j_* representing resource variables *j* = 1, 2…*k* and *β_l_* representing model coefficients *l* = 0, 1 … *k* (McLoughlin et al., 2010).

We defined available points to be within study areas, or “landscapes” (i.e., PP and OMP), and used points to be within home ranges (Dupke et al., 2017; Figure S1). We sampled available points from study areas randomly at 22 points per km^2^. We sampled using points from home ranges in a uniform grid at 70 points per km^2^, using the “spsample” function in the “sp” R package (Bivand et al., 2013). At each point, we extracted the values for white birch quantity carbon (log g/m^2^) and nitrogen composition (%) from our predictive StDM landscape layers. We employed an RSF to compare used and available moose points with explanatory variables being carbon quantity and nitrogen composition and their interaction term. The logistic regression was fit, using the “glm” function (family = binomial, link = logit) in the R statistical program for each study area and its respective seven home ranges and once with data from both study areas and all 14 home ranges.

### 2.5 Forage selection: home range extent

Our next, finer‐scale of foraging was the home range. At this scale, we sought to investigate if areas used by a moose differ to the availability of forage quantities and qualities of its total home range (Johnson, 1980). To do so, we defined available points to be within home ranges, using the same method completed to sample used points in the landscape‐scale analysis. We defined used points to be collar fixes (Dupke et al., 2017; Figure S1). At each point, we extracted the white birch carbon quantity and nitrogen composition measures from our predictive StDM landscape layers. We fit the RSF using a logistic regression with the “glm” function in the R statistical program for each of the 14‐individual moose and once with all individual data from both study areas pooled together. Explanatory variables were carbon quantity and nitrogen composition and their interaction term, like in the landscape scale.

### 2.6 Forage selection: patch extent

The last and most restricted extent of foraging we investigated was the patch in which foraging decisions include the animal's choice of a patch (i.e., 30 m × 30 m pixel) over those available at the time of selection (Charnov, 1976). Here, we adopt an integrated step selection analysis (iSSA) to ask if moose select patches of certain forage quantities or qualities over others (Avgar et al., 2016). The iSSA pairs each used location to a set number of random locations the moose could have viably visited instead based on the distributions of the individual's total step lengths and turn angles (Avgar et al., 2016). This technique of sampling from the animal's natural range of movement speeds, or step lengths, and trajectories, or turn angles, allows for a more precise estimation of fine scale resources available to that animal at a given location.

We performed iSSA with the “amt” R package (Signer et al., 2019). First, we transformed the used fixes into 2‐hr steps (straight line distances between consecutive locations). Prior cleaning of the data created some temporal gaps in between GPS fixes, so we eliminated any steps that had a time difference greater than two hours. A gamma distribution of step lengths (the log transformed value represents the scale parameter) and a von Mises distribution of cosine‐transformed turn angles were used to describe movement behavior (speed and directionality, respectively) of individuals (Avgar et al., 2016). From each start point, 10 available step locations were calculated by randomly extracting step lengths and turn angles from such distributions. We then extracted the white birch carbon quantity and nitrogen composition measures from our predictive StDM landscape layers at all step end locations. Used points were paired to the generated available points in the conditional logistic regressions. Explanatory variables for the model included the quantity carbon values, nitrogen compositions, step lengths, turn angles, and all combinations of interaction terms. We fit the conditional logistic regression model, using the “clogit” function in the R “survival” package, for each of the 14‐individual moose and once with all individual data from both study areas pooled together (Therneau & Grambsch, 2000).

## 3 RESULTS

### 3.1 Descriptive results

The mean predicted quantity carbon of white birch forage from the PP (514 km^2^) and OMP (393 km^2^) study areas were 0.23 g/m^2^ (*SD* = 0.88) and 0.35 g/m^2^ (*SD* = 0.82), respectively. The maximum quantity carbon was 4.09 g/m^2^ in PP and 3.53 g/m^2^ in OMP while minimum values were 0.024 g/m^2^ for PP and 0.021 g/m^2^ for OMP. The mean white birch nitrogen concentration in PP was 2.82% (*SD* = 0.27) and 2.74% (*SD* = 0.28) in OMP. Maximum nitrogen content values were 3.61% and 3.78% and minimum nitrogen contents were 1.89% and 1.59% for the PP and OMP study areas, respectively. The average size of a moose individual's home range for our study's time frame (June 1st–July 16th) was 12.36 km^2^ for PP individuals and 11.07 km^2^ for OMP individuals (Table 2).

**Table 2:** Descriptive statistics (means, medians, standard deviations), and the correlation (Pearson's *r*) for white birch carbon quantity (log g/m^2^) and nitrogen composition (%), from each designated study area and home range

MCP	Sex	Year	Area (km^2^)	Quantity carbon			% Nitrogen			C × N correlation
				Mean	Median	*SD*	Mean	Median	*SD*	Pearson's *r*
PP and OMP	—	—	907	−1.65	−1.67	0.89	2.78	2.78	0.28	−0.01
PP	—	—	514	−1.92	−2.19	0.89	2.82	2.82	0.27	0.11
OMP	—	—	393	−1.41	−1.53	0.82	2.74	2.74	0.28	−0.04
PP2	F	2013	26.27	−1.7	−1.7	0.72	2.77	2.73	0.28	0.03
PP3	M	2011	10.64	−1.87	−1.89	0.7	2.66	2.68	0.29	−0.37
PP4	F	2011	16.28	−2.46	−2.59	0.5	2.78	2.79	0.15	−0.16
PP5	F	2011	22.97	−2.27	−2.31	0.63	2.71	2.72	0.23	0.49
PP6	F	2015	2.12	−1.99	−2.22	0.72	2.63	2.67	0.18	0.59
PP8	F	2011	3.13	−2.08	−2.24	0.7	2.61	2.63	0.15	0.09
PP9	M	2011	5.13	−1.82	−1.65	0.7	3	3	0.14	0.12
OMP4	F	2014	20.28	−1.37	−1.57	0.61	2.79	2.8	0.27	0.13
OMP5	F	2015	10.69	−1.79	−1.76	0.54	2.85	2.86	0.13	0.43
OMP7	M	2014	9.18	−0.8	−0.82	0.83	2.75	2.77	0.26	0.13
OMP11	F	2014	12.06	−1.34	−1.31	0.66	3.04	2.98	0.26	−0.44
OMP12	F	2014	6.68	−1.19	−1.37	0.66	3.03	3	0.26	−0.28
OMP13	F	2015	15.08	−1.74	−1.71	0.96	2.82	2.82	0.18	−0.08
OMP15	M	2014	3.51	−1.36	−1.44	0.83	2.68	2.71	0.19	0.08

Note

For every moose individual, we provide its sex and collar year.

We used selection coefficients from our resource selection analyses to assess the direction and strength of moose selection for white birch quantities and qualities and pseudo *R* ^2^ to assess the strength of our selection analyses. *R* ^2^ *s* from the patch extent iSSAs cannot be directly compared to landscape and home‐range RSFs because of the different model types used (i.e., conditional logistic regression vs. logistic regression). Positive coefficients represent positive selection for the resource, negative coefficients represent avoidance of a resource, and near‐zero coefficients represent neither selection for nor against a resource. Interaction coefficients represent selection trade‐offs between quantity and quality with positive interactions representing a preference for forage quantity over quality and negative interactions representing the reverse. Collectively, we found differing directions and magnitudes of selection for birch quantity and quality, with models showing a wide range of explanatory powers depending on the spatial extent (landscape–home range–patch) and sample‐level (individual or pooled). We did not find year of GPS data to have a significant effect on the model fits (pseudo *R* ^2^ *s*) or observed individual trade‐offs (*f* < 1.10, *p* > .38) for either patch‐level or home range‐level models.

### 3.2 Landscape extent

Selection coefficients for quantity carbon were −0.02 in PP, and −1.03 in OMP. Selection coefficients for nitrogen composition were −1.00 in PP and 2.01 in OMP. Our RSFs explained 1.6% and 3.2% of the variation for PP and OMP, respectively. The pooled model, using data from both study areas had virtually no explanatory power (*R* ^2^ = 0.007) and selection coefficients for quantity carbon and nitrogen composition were −1.66 and 1.32, respectively (Table 3).

**Table 3:** The number of Used (U) and Available (A) points and summaries (intercepts [Int.], *β*‐coefficients, and standard errors [*SE*]) and evaluations (pseudo *R* ^2^) for pooled and individual models measuring moose selection for white birch quantity carbon (Qty C; log g/m^2^) and nitrogen compositions (% N). The landscape (LN) and within‐home range (HR) scales used logistic regressions and conditional logistic regressions were used for the patch (Pt) scale

Scale	Model	U	A	Int.	Qty C		% N		Qty C × % N		*R* ^2^
					*β*	*SE*	*β*	*SE*	*β*	*SE*	
LN	Pooled	8,433	20,000	−4.74	−1.66	0.17	1.32	0.11	0.55	0.06	0.007
LN	PP	4,084	9,345	1.61	−0.02	0.26	−1	0.19	−0.06	0.09	0.016
LN	OMP	4,349	10,655	−6.53	−1.03	0.25	2.01	0.15	0.37	0.09	0.032
HR	Pooled	3,242	8,433	1.76	1.84	0.31	−0.88	0.2	−0.6	0.11	0.006
HR	PP2	171	1,105	2.63	0.29	1.42	−2	1.1	−0.29	0.53	0.026
HR	PP3	310	522	13.79	4.61	1.41	−5.5	0.91	−1.71	0.54	0.18
HR	PP4	230	978	21.05	9.05	2.26	−8.35	2.08	−3.35	0.83	0.012
HR	PP5	124	971	2.74	7.16	2.78	−1.36	1.99	−2.39	0.98	0.083
HR	PP6	210	100	−23.15	−7.46	2.97	8.65	2.34	2.69	1.09	0.076
HR	PP8	67	128	−4.74	−2.55	4.3	1.99	3.02	1.2	1.66	0.04
HR	PP9	240	280	−11.79	−8.41	3.35	4.2	1.99	3.01	1.14	0.048
HR	OMP4	285	1,148	−5.73	−0.31	1.3	1.25	0.75	−0.09	0.44	0.028
HR	OMP5	251	531	38.86	17.01	3.67	−13.34	2.46	−5.67	1.28	0.07
HR	OMP7	343	523	−0.92	0.27	1.03	0.11	0.43	−0.19	0.38	0.009
HR	OMP11	267	671	1.86	2.38	1.32	−0.99	0.79	−0.82	0.43	0.009
HR	OMP12	235	371	4.42	7.31	1.96	−1.48	0.91	−2.24	0.63	0.061
HR	OMP13	361	936	−14.64	−3.7	1.45	4.71	1.02	1.24	0.51	0.051
HR	OMP15	148	169	−16.61	−10.8	3.35	5.45	2.21	3.6	1.21	0.123
Pt	Pooled	2,140	19,242	—	1.11	0.49	−0.14	0.42	−0.32	0.17	0.001
Pt	PP2	90	712	—	7.77	4.712	−1.33	3.59	−2.76	1.65	0.019
Pt	PP3	221	1,930	—	4.69	1.83	−1.51	1.92	−1.69	0.7	0.013
Pt	PP4	145	1,314	—	−4.09	3.73	4.16	4.45	1.79	1.46	0.009
Pt	PP5	74	649	—	7.49	4.07	1.11	3.8	−2.21	1.38	0.016
Pt	PP6	150	1,383	—	−2.4	3.82	11.87	4.06	0.37	1.33	0.022
Pt	PP8	31	225	—	−4.1	7.87	0.21	7.28	1.43	3	0.039
Pt	PP9	160	1,470	—	−1.38	3.49	−0.3	3.14	0.55	1.15	0.01
Pt	OMP4	182	1,632	—	3.57	2.6	0.54	2.1	−1.17	0.84	0.005
Pt	OMP5	145	1,310	—	4.69	4.75	−6.32	4.13	−1.77	1.66	0.006
Pt	OMP7	250	2,362	—	−2.14	1.35	−1.97	1.42	0.86	0.48	0.008
Pt	OMP11	167	1,505	—	0.45	2.11	1.11	2.1	−0.02	0.64	0.009
Pt	OMP12	152	1,305	—	3.45	2.53	−0.81	1.85	−0.93	0.82	0.008
Pt	OMP13	295	2,772	—	−0.37	1.7	3.44	1.8	0.26	0.58	0.009
Pt	OMP15	78	673	—	−5.22	4.79	2.63	4.14	1.44	1.78	0.017

### 3.3 Home range extent

**Figure 3:**
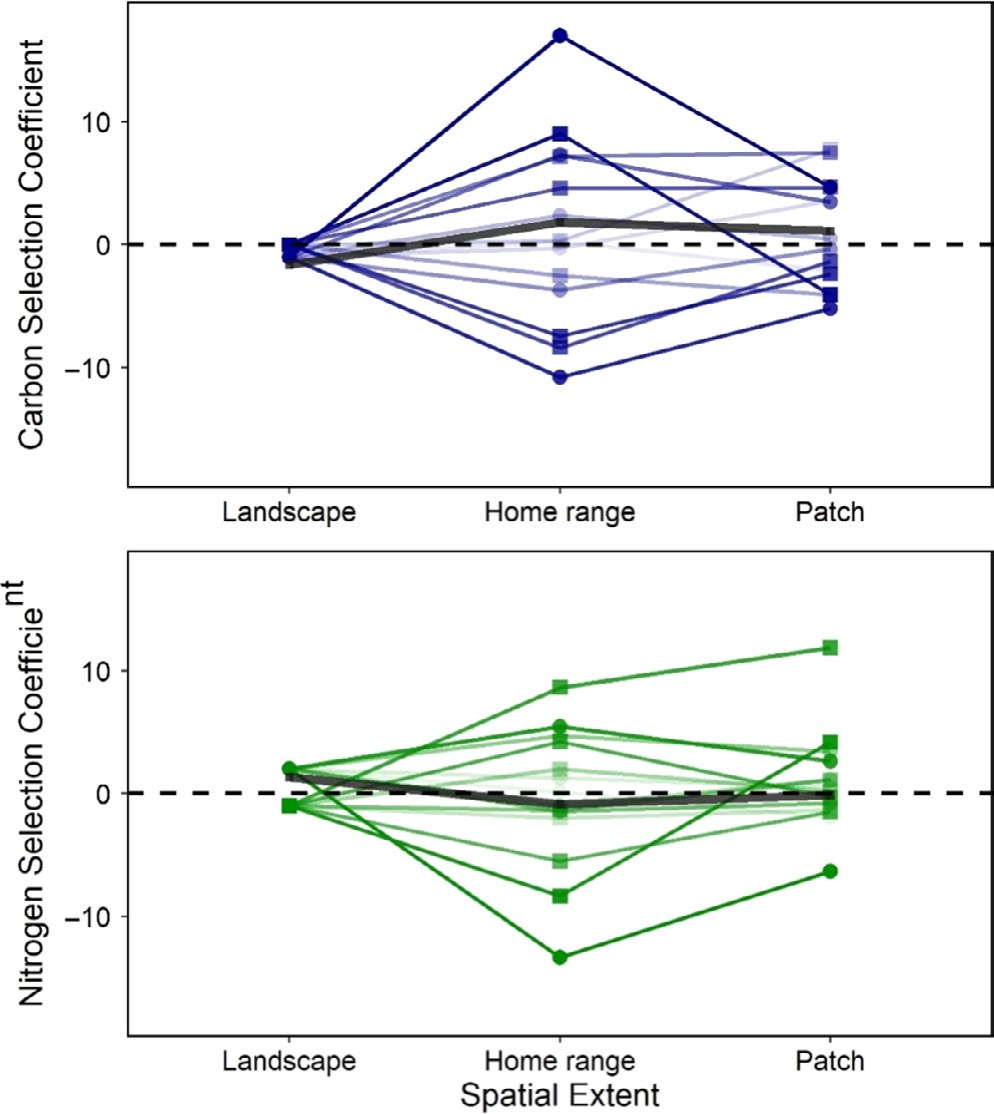
Selection coefficients, positive coefficients representing positive resource selection, for white birch carbon quantities (log g/m^2^) and nitrogen compositions (%) from all three scales of foraging modeled. Individuals’ coefficients are linked between the patch and home‐range scales, and to the coefficient values of their respective study areas (PP = squares, OMP = circles) with lines shaded by the absolute mean of quantity carbon and nitrogen composition coefficients from the individual's home‐range scale models (|Cβ + Nβ|/2). Darker shades represent individuals whose coefficients were, on average, further from zero at the home‐range scale. The black line in each panel shows the coefficients from models using all individuals pooled

**Figure 4:**
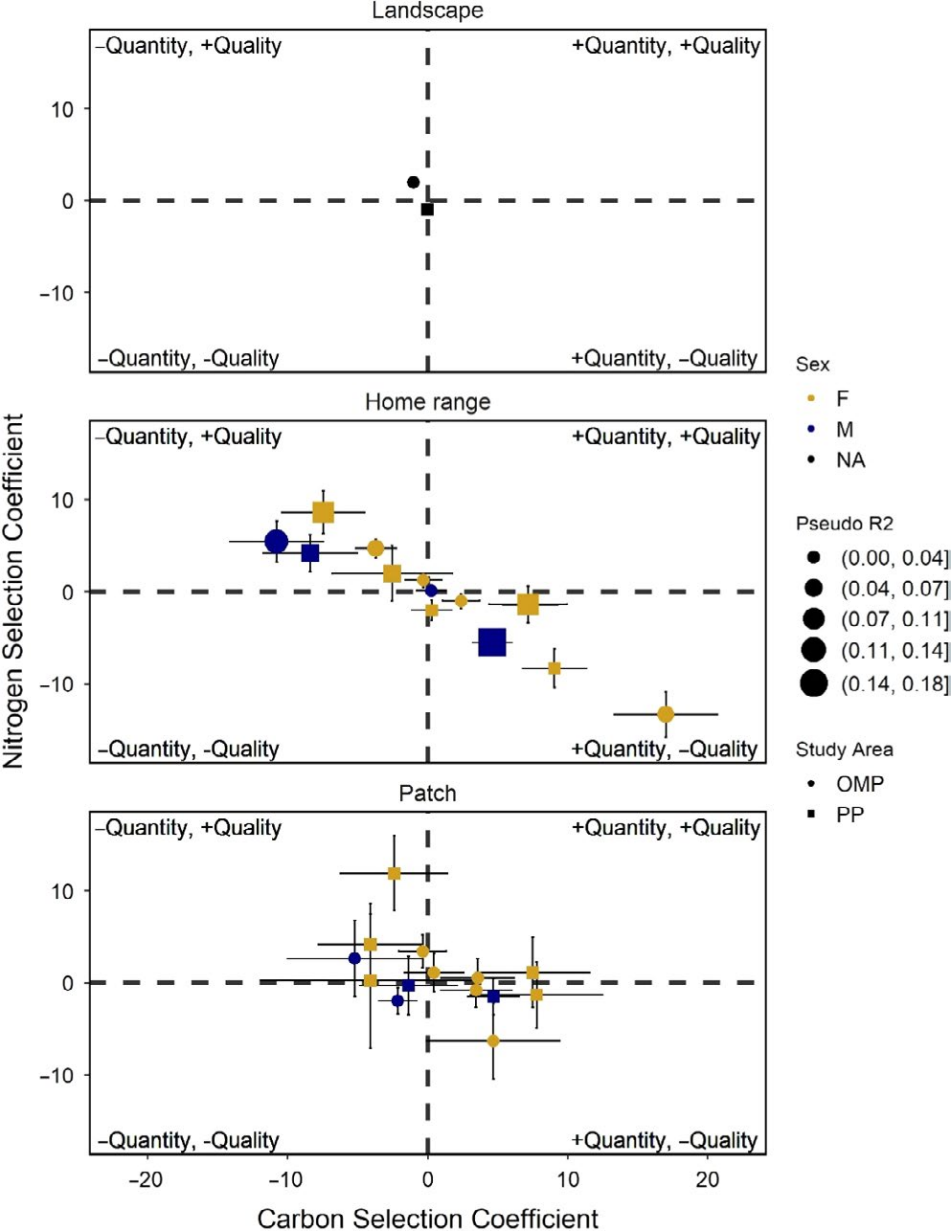
Selection coefficients with standard errors for white birch carbon quantity and white birch nitrogen composition from all scales of foraging modeled in this study, landscape, home range, and patch, plotted against one another. Axis scales are equal across panels, coefficients are scaled in size by their pseudo *R* ^2^, and individuals are distinguished by their study area (shape) and sex (color)

At the home range level is where moose individuals showed the strongest selection. Individuals showed a large range of selection for both forage properties, with carbon selection coefficients ranging from −10.8 to 17.0 and nitrogen selection coefficients ranging from −13.4 to 8.6 (Figure 3, Table 3). Up to 18% of variation was explained in individual models, but some individual models had no explanatory power (pseudo *R* ^2^ 0.009–0.18; Table 3). No individuals negatively selected nor positively selected for both quantity carbon and nitrogen composition; highly positive selection for one component is paired with a negative selection of its counterpart and vice versa (Figure 4). For individual models, pseudo *R* ^2^ *s* did not relate to the number of available points for individual RSFs (*t* = −0.77; *p* = .46). The pooled‐sample model shows weaker selection (Cβ = 1.84, Nβ = −0.88) and virtually no explanatory power (*R* ^2^ = 0.006; Table 3).

### 3.4 Patch extent

Selection coefficients from the patch‐scale iSSAs ranged from −5.22 to 7.77 and −6.32 to 11.87 for white birch quantity carbon and nitrogen composition, respectively (Figure 3; Table 3; Coefficients for step length, turn angle, and their interactions with white birch carbon and nitrogen in Table S1). Individual models explained from zero to 3.9% of selection (pseudo *R* ^2^; Table 3). Similar to the home‐range scale, most individuals did not show simultaneous negative or positive selection for both birch characteristics, but with a somewhat smaller range of coefficient values (Figure 4). For individual models, pseudo *R* ^2^ *s* did not significantly relate to the number of available points for individual RSFs (*t* = −1.95; *p* = .075). The pooled‐sample model shows little to no selection (Cβ = 1.11, Nβ = −0.42) and no explanatory power (*R* ^2^ = 0.001; Table 3).

### 3.5 Interaction coefficients and comparison of scales

**Figure 5:**
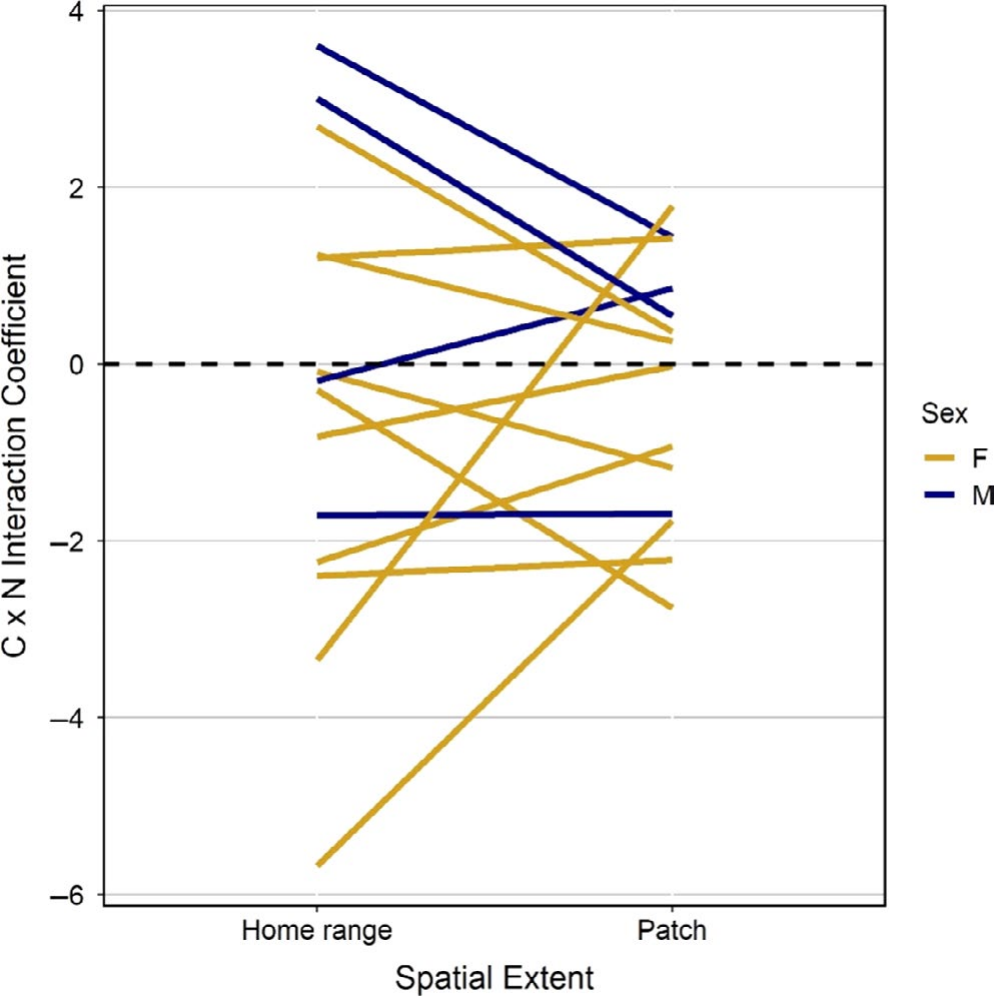
White birch quantity carbon and nitrogen composition interaction coefficients from home range (RSFs) and patch (iSSAs) individual moose selection models. Selection coefficients are linked between scales by individual and colored by individual sex. Positive CxN coefficients represent individuals who selected positively for quantity carbon and negatively for nitrogen compositions, while negative CxN coefficients represent the opposite scenario

The large range of individual coefficients for both carbon and nitrogen measures creates a lack of trend between scale and quantity–quality selection in the pooled models (Figure 3). Within a scale, individual models with the strongest selection trade‐offs (|interaction β‐coefficient|) often had higher explanatory power (Figure 4). Based on the interaction coefficients (positive values representing selection for quantity in avoidance of quality), trade‐off tactics of individuals did not tend to change between the home range and patch extents: carbon–nitrogen interaction coefficients typically converged toward zero with only two individuals switching their trade‐off tactic (Figure 5). We did not find repeated cases of negative correlations between predicted white birch quantity carbon and nitrogen composition from study areas and home ranges (Pearson's *r*; Table 2). These home range white birch correlation values did not have any significant effect on the respective individual model interaction coefficients (linear model weighted by standard error; *t* = −0.523, *p* = .605).

## 4 DISCUSSION

Herbivore foraging strategies reflect the physiological challenge of converting carbon‐heavy matter into more phosphorous and nitrogenous tissues: the tendency to select for plant compositions of higher N and P or plant quantities (Nie et al., 2015). We tested moose resource selection of forage nitrogen content and forage abundance at multiple scales using elemental measures of white birch nitrogen composition (%) and quantity carbon (log g/m^2^), respectively. We found support for our prediction that negative selection for both carbon quantity and nitrogen contents would not occur at any scale, but likewise found no instances of positive selection for both measures, which we predicted would occur at all scales (Figure 4). Unlike findings by van Beest et al. (2010), there was no distinct trend between forage selection tactic and scale (Figure 3). Instead, we found considerable individual variation: at the home‐range scale, individual moose favored either quantity or quality at the expense of the other, with both trade‐off directions expressed at similar magnitudes (Figure 4). Such individual variation should not be overlooked given moose have significant effects on plant biomass and productivity (Ellis & Leroux, 2017), and intraspecific diversity in functional traits can influence total ecosystem processes (Raffard et al., 2019).

At the home‐range scale, we found the strongest selection of white birch nitrogen concentration and biomass (quantity carbon), showing both negative and positive responses for either by individual moose (0 < *R* ^2^ < 0.18). Consequently, we find evidence that moose display distinct quantity–quality trade‐offs within their home ranges (Figure 4). Such trade‐offs support use of birch nitrogen composition as an estimate of forage quality, as has been done in other studies (Ball et al., 2000; Schweiger et al., 2015). Naturally higher nitrogen contents in browse must increase nutrient acquisitions in the digestively constrained moose (Belovsky, 1978), so as to offset their need for prioritizing foraging in areas with high browse abundances. To confirm that trade‐offs in forage selection were not due to growing trade‐off within white birch (i.e., as birch grows it becomes less nitrogenous), we tested for correlation between birch carbon quantity and nitrogen composition across each home range and study area. There were few cases of negative correlations between white birch quantity and quality within home ranges, and furthermore, any white birch StDM correlations did not influence the strength of moose selection trade‐offs. Possibly, a lack of positive correlation between white birch quantities and nitrogen compositions is sufficient to limit moose and force trade‐off foraging strategies. StDMs predict resource elemental compositions, not the allocations of such elements, like PSMs. While nitrogen is most commonly allocated to protein building in plants, tannins can interact with protein– limiting available nitrogen (McArt et al., 2009)—and PSM production by *Betula* can be induced by UV exposure, not necessarily nutrient availability (Keski‐Saari et al., 2005). Thus, environmental driven production of PSMs in white birch could add a layer of complexity to our landscapes of quality. This could potentially explain the diversity of individual selection patterns but would require further plant sampling and landscape modeling to properly investigate.

We found no selection responses at the landscape scale, while patch‐scale models produced coefficients slightly more equivocal than the home‐range models. The lack of selection responses at the landscape scale contradicted our prediction that the landscape‐scale models would result in the highest selection coefficients for birch quantity. Other studies have found that moose display landscape‐level selection for forage quantity as predicted, but when using coarser measures of forage availability (Dussault et al., 2005; Herfindal et al., 2009). Though we predicted that nitrogen composition selection coefficients would be highest at the patch scale, we observed a similar pattern of trade‐offs in the patch scale as the home‐range models (Figure 4). Most individuals maintained their trade‐off tactic from the home range to the patch scale, similar to trade‐offs in roe deer (*Capreolus capreolus*) found by Dupke et al. (2017), but the trade‐offs become more equivocal at the patch scale (Figure 5). This could imply that once a moose selects an area within a home range to forage, the differences between patches may be less important than maintaining high daily forage intake (Belovsky, 1984; Parker et al., 2009). Alternatively, selection may bypass the patch scale and occur within the patch. A study by Astrom et al. (1990) found moose food choice to be better explained at the tree‐level than at the stand‐level, and Danell et al. (1991) also uncovered tree‐level foraging decisions in moose.

Individuals varied the directionality of their quantity and quality selection, overriding any potential sample‐wide trend between spatial extent and selection (Figure 3). Other studies have found herbivory quantity–quality trade‐offs, where all individuals display a similar trade‐off direction (Durant et al., 2004; Van der Wal et al., 2000; Wilmshurst et al., 1999). We find a unique situation in which individuals display opposing trade‐offs, from prioritizing forage quantity over quality, to equal priority for quality over quantity, and many that select for neither. Detecting opposing strategies would not have been possible had our models not been performed at the individual‐level. If moose are indeed plastic in their trade‐off tactic, quantity–quality functional responses remain possible (Leclerc et al., 2016); alternatively, if moose individuals are consistent then fitness should be influenced by trade‐off decisions (Parker et al., 2009; Wam et al., 2018).

Despite having only two predictor variables, we explained anywhere from 0% to 18% of the variation in individual RSFs within forested areas during the early growing season. Rather than use multiple acting landscape variables, such as forest type or aspect ratio, directly in RSAs to infer foraging strategies (Zweifel‐Schielly et al., 2009), we linked these features to plant compositions and biomass first, creating more deterministic, and nutritionally linked resource selection analyses (RSAs; Leroux et al., 2017). Our intent was not to create highly explanatory RSAs across multiple habitats, but rather test how moose select for two specific forage characteristics at multiple scales during a critical window of plant growth in forest patches. Unexplained variation in RSAs could have developed from differences between body masses, sexes, study areas, years (Barboza et al., 2009), the effects of carbon‐based PSMs (Palo, 1984), or tannin‐bound nitrogen (Keski‐Saari et al., 2005). Despite these constraints, we still were able to detect some moose selection for plant nitrogen and biomass, suggesting that our findings are conservative estimates. Ideally, StDMs will be implemented in areas with larger samples of collared animals interpret foraging strategies more confidently.

While nutritional landscapes that predict compound‐based measures of plant quality (e.g., Duparc et al., 2020; Merems et al., 2020) also create deterministic large‐scale foraging analyses, StDMs provide a means to link individual, seasonal, and availability‐dependent differences (Barboza et al., 2009) in herbivore resource selection to carbon and nitrogen cycles. Large terrestrial animals are known to have large‐scale presence–absence or density‐driven effects on plant communities (Estes et al., 2011), yet they are often not incorporated into carbon cycle models (Schmitz et al., 2018). Moose browsing and effects on litter nutritional composition have been shown to negatively impact ecosystem net primary productivity (Ellis & Leroux, 2017; Schmitz et al., 2014). The individuals of this study that chose to forage in areas of higher birch carbon quantities rather than areas of higher nitrogenous birch could be the individuals which have, directly, the largest negative effect on plant productivity (Kolstad et al., 2018). We also interpret our observed trade‐off as evidence that moose may strive to meet certain nitrogen intake amounts (0.627 ± 0.073 g/kg BW/day; (Schwarts et al., 1987)) by consuming larger quantities or food of higher nitrogen contents. Daily nitrogen intake not only equals the nitrogen removal from primary producers, it also positively correlates to fecal nitrogen content (Howery & Pfister, 1990), which could be integrated into nitrogen cycling models. With moose being a dominant browser across the boreal biome, their foraging behaviors can have implications to boreal zoogeochemistry (Schmitz et al., 2018).

Connecting ecological theory across scales and systems has remained a problem in ecology (Levin, 1992). Scale presents particular challenges for ungulate foraging, because such species react to plant distributions from the bite to the regional‐level, making a case for tools like StDMs that capture the heterogeneity of plant qualities across landscapes (Leroux et al., 2017; Weisberg & Bugmann, 2003). Further, the use of elemental currencies in foraging studies creates findings that can be compared across systems. Ungulates can rapidly change plant communities, nutrient cycles, and whole ecosystems through herbivory and fecal deposition (Didion et al., 2009; Hobbs, 2018). In our study we found that some moose in a population make individually varying trade‐offs between both forage quantity and quality, implying that moose are nutritionally limited but flexible in their intake tactics. With the current accessibility of remote sensing data and wildlife monitoring technology, we have the opportunity to make inferences about animal responses to fine‐scaled, biogeochemical processes.

## CONFLICT OF INTEREST

We do not have any conflicts of interest to report.

## AUTHOR CONTRIBUTION

**Juliana Balluffi‐Fry:** Conceptualization (lead); Formal analysis (lead); Visualization (lead); Writing‐original draft (lead); Writing‐review & editing (equal). **Shawn James Leroux:** Conceptualization (equal); Data curation (equal); Funding acquisition (equal); Investigation (equal); Methodology (equal); Project administration (equal); Supervision (supporting); Writing‐review & editing. **Yolanda F. Wiersma:** Conceptualization (equal); Data curation (equal); Funding acquisition (equal); Investigation (equal); Methodology (equal); Project administration (equal); Supervision (supporting); Writing‐review & editing (equal). **Travis Heckford:** Conceptualization (equal); Investigation (equal); Methodology (equal); Validation (equal); Writing‐review & editing (equal). **Matteo Rizzuto:** Conceptualization (equal); Investigation (equal); Methodology (equal); Validation (equal); Writing‐review & editing (equal). **Isabella Croft Richmond:** Conceptualization (equal); Investigation (equal); Methodology (equal); Validation (equal); Writing‐review & editing (equal). **Eric Vander Wal:** Conceptualization (equal); Data curation (equal); Formal analysis (equal); Funding acquisition (equal); Investigation (equal); Methodology (equal); Project administration (equal); Supervision (lead); Visualization (supporting); Writing‐original draft (supporting); Writing‐review & editing (equal).

## Supporting information

Appendix S1

## Data Availability

Cleaned data and code for resource selection analyses are available on GitHub at https://github.com/jballuffi/MooseForagingStoichiometry and on Dryad at https://doi.org/10.5061/dryad.crjdfn32x.
