# Supplementary material for: Quantity–quality trade‐offs revealed using a multiscale test of herbivore resource selection on elemental landscapes
Source: Ecol Evol. 2020 Nov 18;10(24):13847–59. doi: 10.1002/ece3.6975 (PMC7771173; doi:10.1002/ece3.6975)
Supplement: Supplementary file 1 — Appendix S1 [file ECE3-10-13847-s001.docx]

$$\left( \right){}{}{}{}{}{}{}$$

Appendix for:

**Quantity-quality trade-offs revealed using a multiscale test of herbivore resource selection on elemental landscapes**

Juliana Balluffi-Fry*, Shawn J. Leroux, Yolanda F. Wiersma, Travis R. Heckford, Matteo Rizzuto, Isabella C. Richmond and Eric Vander Wal

*Department of Biology, Memorial University of Newfoundland, St. John’s, Canada*

*Corresponding author: Juliana Balluffi-Fry; Department of Biology, Memorial University of Newfoundland, 230 Elizabeth Avenue, St. John’s, Canada; email: jballuffifry@mun.ca

Total appendices: 3

Number of tables: 1

Number of figures: 2

Total word count: 613

Table A1. Summaries (β-coefficients and standard errors) of additional terms in the patch-scale integrated step selection functions which are fit with conditional logistic regressions. Terms include step lengths (SL), turn angles (TA), and their interactions between the other, and with carbon quantities (C), nitrogen concentrations (N). For each moose individual we also provide its mean step length (SL; meters) and mean turn angle (TA; degrees) from used steps.

|  |  |  |  | SL | | TA | | C x SL | | N x SL | | C x TA | | N x TA | | SL x TA | |
| --- | --- | --- | --- | --- | --- | --- | --- | --- | --- | --- | --- | --- | --- | --- | --- | --- | --- |
| ID | **SL** | **TA** | **R²** | **β** | **SE** | **β** | **SE** | **β** | **SE** | **β** | **SE** | **β** | **SE** | **β** | **SE** | **β** | **SE** |
| Pooled | 189.14 | 0.81 | 0.001 | 0.22 | 0.18 | -0.8 | 0.35 | -0.04 | 0.02 | -0.11 | 0.06 | 0.09 | 0.04 | 0.28 | 0.12 | 0.04 | 0.02 |
| PP2 | 187.32 | 14.64 | 0.019 | 0.98 | 0.9 | -0.23 | 2.16 | -0.14 | 0.15 | -0.49 | 0.33 | 0 | 0.33 | -0.15 | 0.78 | 0.19 | 0.11 |
| PP3 | 199.48 | -1.95 | 0.013 | 0.92 | 0.59 | -1.72 | 1.11 | -0.06 | 0.12 | -0.39 | 0.29 | 0.6 | 0.21 | 1.16 | 0.51 | -0.04 | 0.08 |
| PP4 | 247.88 | 2.6 | 0.009 | -1.27 | 1.12 | 0.37 | 2.53 | -0.11 | 0.18 | 0.34 | 0.43 | 0.55 | 0.41 | 0.65 | 0.99 | -0.13 | 0.08 |
| PP5 | 227.14 | -14.82 | 0.016 | 2.02 | 1.86 | 0.8 | 3.63 | -0.23 | 0.16 | -0.8 | 0.64 | -0.33 | 0.29 | -0.67 | 1.16 | 0.12 | 0.13 |
| PP6 | 61.79 | -18.45 | 0.022 | 6.41 | 2.31 | 9.85 | 3.81 | 0.15 | 0.16 | -2.24 | 0.75 | 0.91 | 0.26 | -2.85 | 1.21 | -0.18 | 0.11 |
| PP8 | 177.39 | 18.05 | 0.039 | 0.32 | 2.99 | 12.48 | 5.97 | 0.15 | 0.19 | 0.07 | 1.15 | -0.14 | 0.41 | -4.44 | 2.15 | -0.21 | 0.19 |
| PP9 | 126.3 | 5.72 | 0.01 | 0.21 | 1.67 | -3.02 | 2.73 | 0.01 | 0.07 | -0.08 | 0.55 | 0.1 | 0.12 | 1 | 0.89 | 0.09 | 0.09 |
| OMP4 | 224.63 | 4.78 | 0.005 | 1 | 0.79 | -2.56 | 1.78 | -0.04 | 0.13 | -0.38 | 0.27 | 0.2 | 0.26 | 0.85 | 0.59 | 0.08 | 0.07 |
| OMP5 | 198.72 | -2.28 | 0.006 | -0.72 | 1.5 | -3.25 | 2.8 | 0.1 | 0.16 | 0.32 | 0.49 | -0.43 | 0.26 | 0.86 | 0.94 | -0.01 | 0.09 |
| OMP7 | 231.82 | 9.8 | 0.008 | -0.6 | 0.66 | -1.58 | 1.34 | -0.03 | 0.07 | 0.21 | 0.24 | 0.26 | 0.13 | 0.47 | 0.47 | 0.13 | 0.07 |
| OMP11 | 287.33 | -5.62 | 0.009 | -0.02 | 0.82 | -5.55 | 1.66 | -0.07 | 0.13 | -0.03 | 0.3 | 0.01 | 0.24 | 1.68 | 0.57 | 0.09 | 0.09 |
| OMP12 | 117.77 | 1.45 | 0.008 | 0.05 | 0.98 | 3.23 | 1.77 | -0.04 | 0.1 | -0.03 | 0.33 | 0.05 | 0.19 | -1 | 0.59 | -0.02 | 0.08 |
| OMP13 | 209.32 | -12.63 | 0.009 | 0.77 | 0.72 | -3.29 | 1.44 | -0.09 | 0.05 | -0.34 | 0.24 | 0.15 | 0.09 | 1.15 | 0.47 | 0.08 | 0.06 |
| OMP15 | 151.04 | 9.99 | 0.017 | -0.29 | 1.15 | -1.11 | 2.37 | 0.12 | 0.2 | 0.18 | 0.42 | 0.41 | 0.36 | 0.66 | 0.89 | -0.01 | 0.12 |


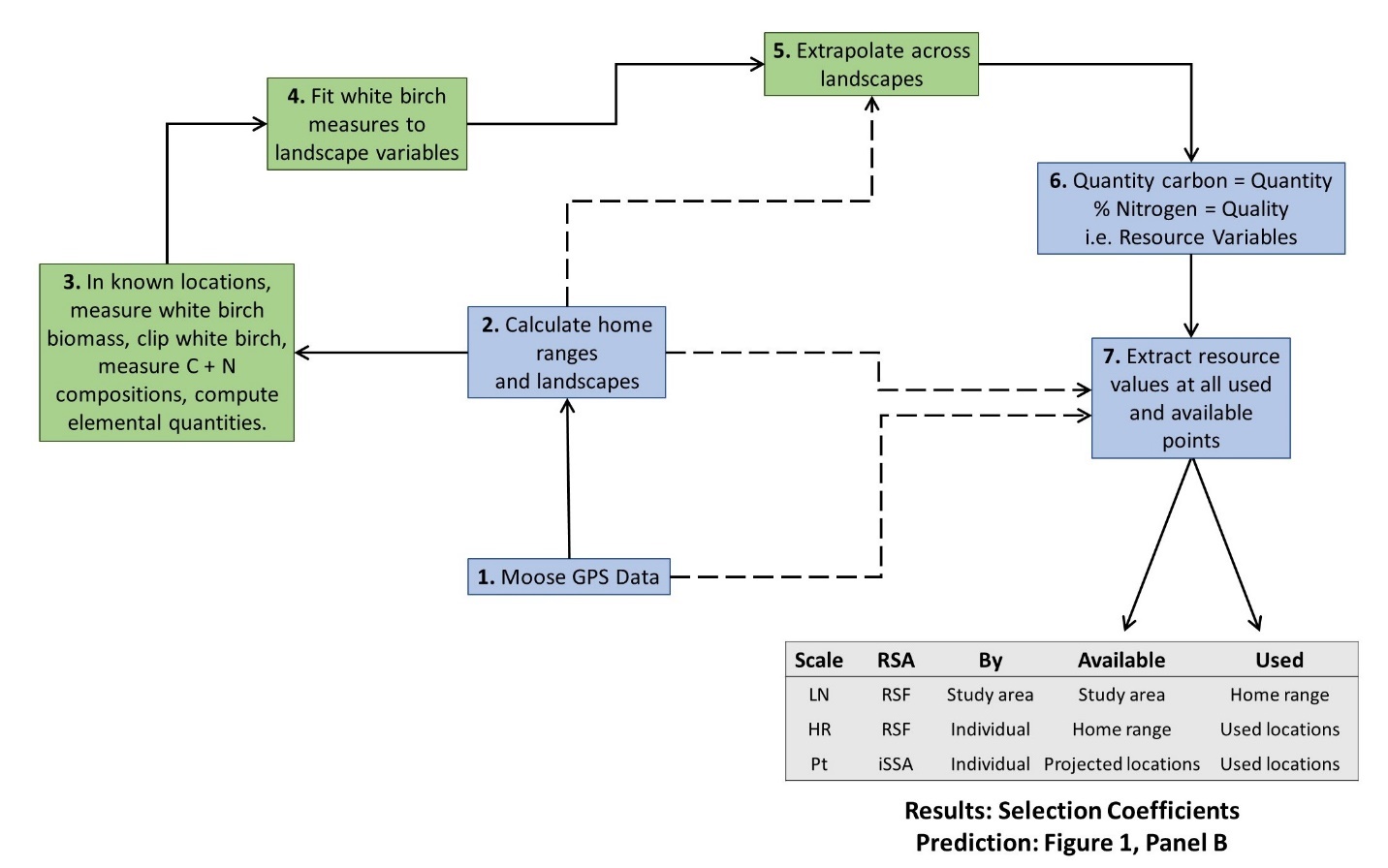


Figure A1. A diagram mapping the analysis pathway to testing the predictions of Figure 1, Panel B, using collar data and Stoichiometric Distribution Models (StDMs). Green boxes represent StDM steps. Blue boxes represent steps that created the data which were directly incorporated into the resource selection analyses (RSAs). Solid lines represent the sequence of direct steps in the total analysis, and dashed lines represent non-sequential input of steps. We used moose GPS data to calculate home ranges and landscapes, from within we sampled forage for stoichiometric measurements to then model and extrapolate across the respective landscapes. The forage measures for quantity and quality are extracted from GPS data and GPS data derived spatial extents (i.e. landscapes or home ranges) and locations (i.e. projected locations). Lastly, we ran models using appropriate data for each scale of foraging.


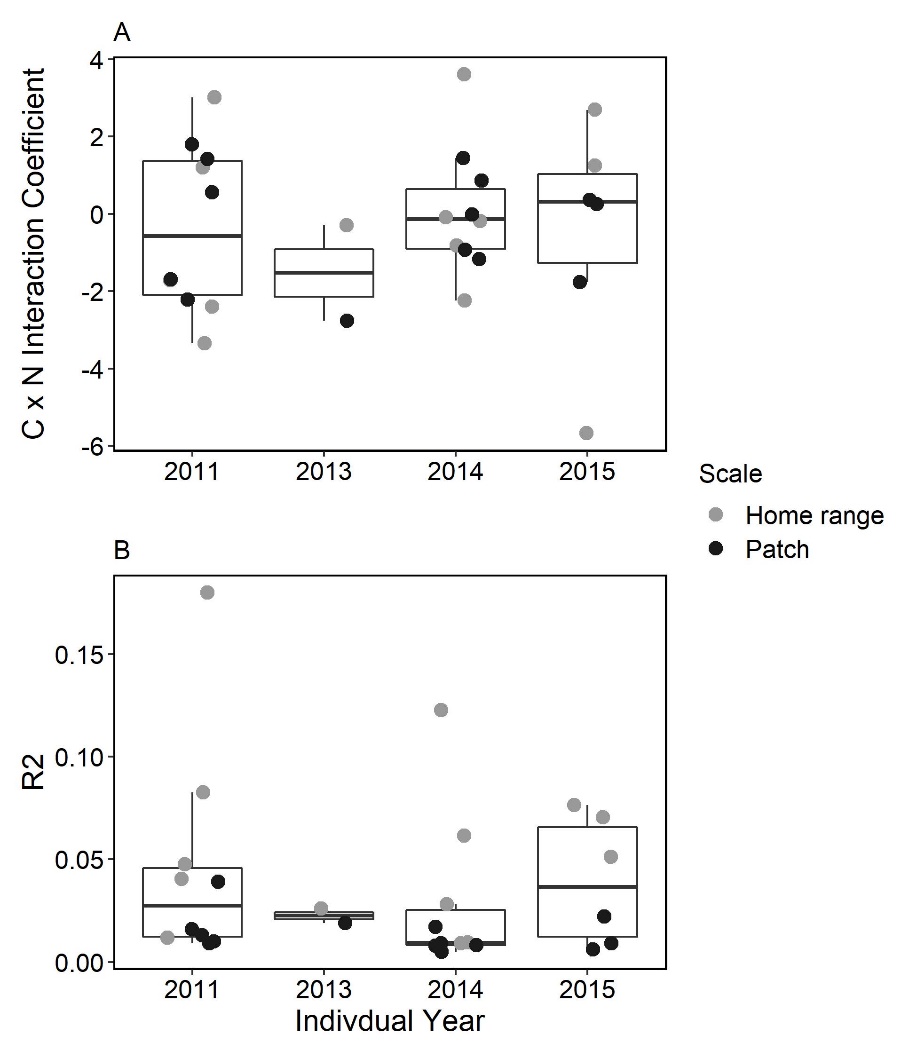


Figure A2. Interaction coefficients (A) and pseudo R²s (B) from individual moose selection models at the home range (grey; RSFs) and patch (iSSAs; black) scales compared to the year GPS data was collected for the individuals. Models measured moose selection for white birch quantity carbon and nitrogen compositions that were calculated using Stoichiometric Distribution Models based on ground-collected samples from 2015. Interaction coefficients represent the quantity-quality selection trade-off made by moose. We did not find year of GPS data to have a significant effect on the model fits (pseudo R²s) or observed individual trade-offs (p > 0.38) for either patch-level or home range-level models.
